# COVID-19 pandemic-related healthcare interruptions and diabetes distress: a national study of US adults with diabetes

**DOI:** 10.1186/s12889-024-17921-3

**Published:** 2024-02-16

**Authors:** Ankeeta Saseetharran, Shivani A. Patel

**Affiliations:** Emory Rollins School of Public Health, 1518 Clifton Rd, 30322 Atlanta, GA USA

**Keywords:** Diabetes distress, COVID-19 pandemic, Type 1 diabetes, Type 2 diabetes

## Abstract

**Background:**

Early COVID-19 pandemic research found changes in health care and diabetes management, as well as increased diabetes distress. This study aims to determine the association between COVID-19 pandemic-related healthcare interruptions and diabetes distress among adults with Type 1 and Type 2 diabetes in the US in 2021.

**Methods:**

Multinomial logistic regression was used to analyze moderate and high levels of diabetes distress (reference = no diabetes distress) in 228 individuals with Type 1 diabetes and 2534 individuals with Type 2 diabetes interviewed in the National Health Interview Survey in 2021.

**Results:**

Among adults with Type 1 diabetes, 41.2% experienced moderate diabetes distress and 19.1% experienced high diabetes distress, and among adults with Type 2 diabetes, 40.8% experienced moderate diabetes distress and 10.0% experienced high diabetes distress. In adults with Type 1 diabetes, experiencing delayed medical care was associated with an adjusted odds ratio (aOR) of 4.31 (95% CI: 1.91–9.72) for moderate diabetes distress and 3.69 (95% CI: 1.20–11.30) for high diabetes distress. In adults with Type 2 diabetes, experiencing delayed medical care was associated with an aOR of 1.61 (95% CI: 1.25–2.07) for moderate diabetes distress and 2.27 (95% CI: 1.48–3.49) for high diabetes distress. Similar associations were observed between not receiving medical care due to the pandemic and diabetes distress. Conclusion: Among people with diabetes, experiencing delayed medical care and not receiving care due to the pandemic were associated with higher reports of diabetes distress.

**Supplementary Information:**

The online version contains supplementary material available at 10.1186/s12889-024-17921-3.

## Background

Diabetes distress is the worry, conflict, frustration, and discouragement that can accompany living with diabetes [1]. It poses a psychological burden on patients and is linked to poorer diabetes management. Diabetes distress is associated with self-efficacy and diabetes self-care practices, such as medication adherence, foot care, diet, exercise, and glucose self-monitoring [1, 2]. Diabetes distress has been observed to be associated with poor glycemic control, lower medication adherence, and increased missed insulin boluses [2–6]. In turn, suboptimal control or uncontrolled diabetes can result in serious complications, such as kidney disease, retinopathy, neuropathy, and nephropathy, as well as life-threatening difficulties [7].

Healthcare access has been linked to diabetes distress. Healthcare access includes the ability to engage with healthcare providers, quality of interactions with healthcare providers, and the ability to navigate the healthcare system [8–11]. Adults with easier access to healthcare providers and better healthcare provider support report less diabetes distress [8, 9, 12]. During the pandemic, interruptions and changes to health care and diabetes management have been documented among individuals with Type 1 and Type 2 diabetes [13]. Particularly in the first few weeks of the pandemic, approximately 10% of respondents in a national study reported difficulties contacting their health care team, and approximately 30% reported cancelled or postponed labs and tests. Over half of these respondents reported an increase in diabetes distress at the onset of the pandemic [14].

It is unclear, however, whether the interruptions to healthcare and related distress at the onset of the pandemic persisted in subsequent years when vaccines became available, social distancing restrictions had begun loosening, and thus pandemic-related constraints on healthcare access lessened. To address this gap, we investigated the extent to which pandemic-related healthcare interruptions were reported by people with Type 1 and Type 2 diabetes in the United States in 2021, and whether there was an association between pandemic-related healthcare interruptions and diabetes distress.

## Methods

### Data source

Data were obtained from the National Health Interview Survey (NHIS) from 2021. This is a cross-sectional household interview survey conducted by the National Center for Health Statistics at the Centers for Disease Control and Prevention. The study population consists of participants from the civilian noninstitutionalized US population in the 50 states and District of Columbia, excluding those who reside in long term care institutions, correctional facilities, and foreign countries. Geographically clustered sampling techniques were used to select a nationally representative sample of dwelling units. Interviews were conducted both in-person and by phone, and data were collected continuously from January to December 2021 [15]. Adults aged 20 years and older with either Type 1 diabetes (*n* = 249) or Type 2 diabetes (*n* = 2647) were eligible to be included in this study. The analysis was restricted to individuals who had complete information on exposures, outcomes, and study covariates (228 individuals with Type 1 diabetes and 2534 individuals with Type 2 diabetes).

### Diabetes distress

The primary outcome was diabetes distress, which was queried in the NHIS survey as a single global item measuring how often the individual felt overwhelmed by the demands of living with diabetes in the past month. Participants responded “always”, “usually”, “sometimes”, “rarely”, or “never.” For the analysis, the outcome was categorized into three groups: high distress (“always” or “usually”), moderate diabetes distress (“sometimes” or “rarely”), or no diabetes distress (“never”). A three-level classification was chosen to align with prior literature suggesting that high, moderate, and little or no diabetes distress are meaningful groupings in relation to diabetes outcomes [16].

### Pandemic-related healthcare interruptions

Primary exposures were pandemic-related healthcare interruptions, which included delayed medical care and not receiving medical care. Participants were asked if there was a time when they delayed getting medical care because of the COVID-19 pandemic or if there was a time when they needed medical care for something other than COVID-19, but did not get it because of the COVID-19 pandemic. These exposures were binary with “yes” or “no” responses.

### Demographic and health covariates

Covariates included in the model were age, sex, race and ethnicity, marital status, geographical region, educational level, employment status, income, health insurance status, anxiety, and depression. Previous literature shows that diabetes distress is associated with anxiety, depressive symptoms, and diabetes complications [1, 4, 5, 17–23]. Higher diabetes distress levels are significantly associated with younger age, lower income, non-white race/ethnicity, female gender, and higher blood glycemic levels [5, 19, 23–27]. Several studies on subsets of populations with either Type 1 or Type 2 diabetes suggest that there are differences in healthcare support, utilization, and access by age, sex, race/ethnicity, income, health insurance status, depression, and education [9, 26, 28–32]. Individuals satisfied with their diabetes care reported lower anxiety scores [33]. Literature also suggests that employment status, geographic region, education, and marital status could also be potential confounders in looking at the association between health care and diabetes distress [12, 33–36].

Age was a continuous variable from 20 to 84 years old. Sex was self-reported as male or female. Combined race and ethnicity were categorized as Hispanic, non-Hispanic white, non-Hispanic black, and other. Marital status was dichotomized as “currently married or cohabiting” and “other”. Geographical region was dichotomized as “metropolitan” for individuals reporting living in large central metros, large fringe metros, or medium and small metros, and “non-metropolitan” if individuals lived outside of a metropolitan area. Educational level was categorized as below high school, high school graduate/GED/equivalent, and college or above. Current employment status was determined from a “yes” or “no” answer to the question of whether or not the individual worked the previous week. Income was the ratio of family income to poverty threshold for the sampled adult’s family grouped into the following categories: 0-0.99, 1-1.99, 2-2.99, and 3 or greater. Health insurance status was reported as “yes” or “no” in response to the question of whether or not the individual was covered by any kind of health insurance or health care plan. Anxiety was self-reported as ever being told by a doctor or health professional that they have an anxiety disorder, and depression was self-reported as ever being told by a doctor or health professional that they have any type of depression.

### Statistical analysis

All analyses accounted for the survey design using sampling elements and weights provided by NHIS.

Descriptive analysis was conducted to determine the prevalence of background characteristics, exposures, and outcomes. Multinomial logistic regression was conducted for individuals with Type 1 diabetes and Type 2 diabetes separately. One set of multinomial logistic regression models evaluated the unadjusted associations between each of the pandemic-related healthcare interruption variables and diabetes distress specified as a three-level variable (reference = no report of diabetes distress). A second set of multinomial logistic regression models evaluated the adjusted associations between each exposure and the outcome. The adjusted models included age, sex, race and ethnicity, marital status, geographical region, educational attainment, employment status, income, health insurance status, anxiety, and depression as they were identified as potential confounders from previous literature. Furthermore, we assessed whether the association between pandemic-related healthcare interruptions and diabetes distress was modified by the type of diabetes by including the presence of statistical interaction between the presence of the healthcare exposure and type of diabetes in a model pooling individuals with Type 1 and Type 2 diabetes.

A sensitivity analysis was conducted to assess whether findings were robust to the three-level categorization of diabetes distress. Whereas the primary analysis modeled diabetes distress as a three-level outcome, the sensitivity analysis modeled diabetes distress as a five-level categorical variable: always, usually, sometimes, rarely, and never.

## Results

### Descriptive analysis

Characteristics of US adults with diabetes in 2021 based on a nationally representative survey sample are provided in Table 1. Individuals with Type 1 diabetes had a mean age of 51.9 ± 1.3 years old. They self-reported as 59.3% non-Hispanic white, 14.7% non-Hispanic black, and 18.4% Hispanic. 56.2% reported an educational attainment of college or above, 50.6% were employed as of the previous week, and 96.9% had health insurance. 82.8% reported living in a metropolitan area and 57.0% were married or cohabiting with a partner (Table 1).

Individuals with Type 2 diabetes had a mean age of 62.8 ± 0.3 years old. They self-reported as 58.0% non-Hispanic white, 16.0% non-Hispanic black, and 17.7% Hispanic. 50.2% reported an educational attainment of college or above, 38.5% were employed as of the previous week, and 95.7% had health insurance. 84.0% reported living in a metropolitan area and 63.8% were married or cohabiting with a partner (Table 1).

**Table 1:** Characteristics of adults 20 years and older with Type 1 and Type 2 diabetes in the US, NHIS 2021

	Type 1 diabetes (*n*=228)	Type 2 diabetes (*n*=2534)
Population size (weighted frequency)	1,951,899	19,350,659
Age in years, mean ± SD^2^	51.9 ± 1.3	62.8 ± 0.3
Female, %	47.0 (39.4-54.7)	48.5 (46.1-50.8)
Race and ethnicity, %		
Non-Hispanic White Non-Hispanic Black/African American	59.3 (51.3-67.2) 14.7 (8.9-20.5)	58.0 (55.1-60.9) 16.0 (14.0-18.1)
Hispanic	18.4 (12.0-24.8)	17.7 (15.2-20.2)
Other (non-Hispanic Asian, non-		
Hispanic AIAN^3^, multiracial, other)	7.6 (2.5-12.6)	8.3 (6.7-9.8)
Married or cohabiting, %	57.0 (49.5-64.4)	63.8 (61.6-65.9)
Lives in a metropolitan area, %	82.8 (77.2-88.5)	84.0 (82.0-86.0)
Educational attainment, %		
Below high school	15.7 (10.1-21.3)	16.4 (14.5-18.3)
High school graduate, GED, or equivalent	28.1 (21.2-35.0)	33.5 (31.2-35.8)
College or above	56.2 (48.7-63.8)	50.2 (47.8-52.5)
Employed, %	50.6 (43.1-58.1)	38.5 (36.3-40.8)
Family income to poverty, %		
0-0.99	13.4 (8.4-18.3)	11.4 (9.9-12.9)
1.00-1.99	21.9 (15.3-28.4)	23.1 (21.2-25.1)
2.00-2.99	17.0 (11.4-22.6)	20.0 (18.1-22.0)
3.00 or greater	47.8 (40.5-55.1)	45.4 (43.0-47.8)
Has health insurance, %	96.9 (93.9-99.8)	95.7 (94.6-96.7)
Ever had anxiety disorder, %	24.1 (17.8-30.4)	19.8 (18.0-21.6)
Ever had depression, %	29.8 (22.9-36.7)	23.9 (21.9-25.9)

^1^ *Table cell values are given as percents (95% confidence interval) unless otherwise indicated. Prevalence point estimates and 95% confidence intervals are presented to convey precision of estimates in the population of adults with Type 1 or Type 2 diabetes in the United States*

^*2*^ *SD = standard deviation*

^*3*^ *AIAN = American Indian, Alaska Native*

41.2% of individuals with Type 1 diabetes and 40.8% of individuals with Type 2 diabetes reported experiencing moderate diabetes distress, while 19.1% of individuals with Type 1 diabetes and 10.0% of individuals with Type 2 diabetes reported experiencing high diabetes distress. Individuals with Type 1 diabetes reported experiencing a statistically significantly higher prevalence of moderate or high levels of diabetes distress than individuals with Type 2 diabetes (*p* < 0.001). 23.2% of individuals with Type 1 diabetes and 21.1% of individuals with Type 2 diabetes reported experiencing delayed medical care. 18.0% of individuals with Type 1 diabetes and 15.1% of individuals with Type 2 diabetes reported not receiving medical care. (Table 2).

**Table 2:** Prevalence of pandemic-related healthcare interruptions and diabetes distress experienced among adults 20 years and older with Type 1 and Type 2 diabetes in the US, NHIS 2021

	Type 1 diabetes (*n* = 228) Prevalence (95% CI)	Type 2 diabetes (*n* = 2534) Prevalence (95% CI)	p-value comparing difference between Type 1 and Type 2 diabetes
Experienced delayed medical care	23.2 (17.3–29.2)	21.1 (19.2–23.1)	*p* = 0.50
Did not get medical care	18.0 (12.7–23.3)	15.1 (13.5–16.7)	*p* = 0.28
Diabetes distress			*p* < 0.001
None	39.7 (31.9–47.4)	49.2 (46.7–51.6)	
Moderate	41.2 (33.6–48.9)	40.8 (38.3–43.3)	
High	19.1 (13.8–24.5)	10.0 (8.6–11.5)	

^1^Prevalence point estimates and 95% confidence intervals are presented to convey precision of estimates in the population of adults with Type 1 or Type 2 diabetes in the United States

### Associations between pandemic-related healthcare interruptions and diabetes distress

Among adults with Type 1 diabetes (Table 3), the unadjusted odds of moderate diabetes distress in those who reported experiencing delayed medical care was 4.21 (95% CI:1.85–9.58) times the odds of moderate diabetes distress among those who did not report experiencing delayed medical care. The unadjusted odds of high diabetes distress in those who reported experiencing delayed medical care was 4.17 (95% CI: 1.68–10.32) times the odds of high diabetes distress among those who did not report experiencing delayed medical care. The unadjusted odds of moderate diabetes distress among those who reported missed medical care was 3.21 (95% CI:1.26–8.15) times the odds of moderate diabetes distress among those who did not report missed medical care. The unadjusted odds of high diabetes distress in those who reported experiencing missed medical care was 3.76 (95% CI: 1.40-10.09) times the odds of high diabetes distress among those who did not report experiencing missed medical care. After adjusting for age, sex, race and ethnicity, education, income, health insurance status, marital status, employment status, geographical region, anxiety, and depression, experiencing delayed medical care was associated with an odds ratio of 4.31 (95% CI: 1.91–9.72) for moderate diabetes distress and 3.69 (95% CI: 1.20–11.30) for high diabetes distress. Similarly, experiencing missed medical care was associated with an odds ratio of 3.41 (95% CI: 1.31–8.87) for moderate diabetes distress and 3.07 (95% CI: 0.82–11.50) for high diabetes distress.

Among individuals with Type 2 diabetes (Table 3), the unadjusted odds of moderate diabetes distress in those who reported experiencing delayed medical care was 1.72 (95% CI:1.35–2.19) times the odds of moderate diabetes distress among those who did not report experiencing delayed medical care. The odds of high diabetes distress in those who reported experiencing delayed medical care was 2.60 (95% CI: 1.75–3.86) times the odds of high diabetes distress among those who did not report experiencing delayed medical care. The odds of moderate diabetes distress among those who reported missed medical care was 1.54 (95% CI: 1.67–2.03) times the odds of moderate diabetes distress among those who did not report missed medical care. The odds of high diabetes distress among those who reported missed medical care was 2.65 (95% CI: 1.73–4.06) times the odds of high diabetes distress among those who did not report missed medical care. After adjusting for sociodemographic and psychosocial characteristics, experiencing delayed medical was associated with an aOR of 1.61 (95% CI: 1.25–2.07) for moderate diabetes distress and 2.27 (95% CI: 1.48–3.49) for high diabetes distress. Similarly, experiencing missed medical care was associated with an aOR of 1.37 (95% CI: 1.03–1.83) for moderate diabetes distress and 2.08 (95% CI: 1.27–3.39) for high diabetes distress.

Tests for interaction between exposures shown in Table 3 and diabetes type indicated that the association between pandemic-related healthcare interruptions and diabetes distress was substantially stronger in individuals with Type 1 compared to individuals with Type 2 diabetes (*p* < 0.001) (data not shown).

**Table 3:** Associations of pandemic-related healthcare interruptions and diabetes distress among adults with Type 1 and 2 diabetes in the US, NHIS 2021

		Adults with Type 1 diabetes (*n* = 228) OR (95% CI; p-value)		
**Exposure**		No Diabetes Distress	Moderate Diabetes Distress	High Diabetes Distress
Reported delayed medical care*	Unadjusted	Ref	4.21 (1.85–9.58; *p* < 0.001)	4.17 (1.68–10.32; *p* = 0.002)
	Adjusted^1^	Ref	4.31 (1.91–9.72; *p* < 0.001)	3.69 (1.20–11.30; *p* = 0.020)
Reported not receiving medical care**	Unadjusted	Ref	3.21 (1.26–8.15; *p* = 0.010)	3.76 (1.40-10.09; *p* = 0.009)
	Adjusted^1^	Ref	3.41 (1.31–8.87; *p* = 0.010)	3.07 (0.82–11.50; *p* = 0.100)
		**Adults with Type 2 diabetes** (*n* = **2534)** OR (95% CI; p-value)		
Reported delayed medical care*	Unadjusted	Ref	1.72 (1.35–2.19; *p* < 0.001)	2.60 (1.75–3.86; *p* < 0.001)
	Adjusted^1^	Ref	1.61 (1.25–2.07; *p* < 0.001)	2.27 (1.48–3.49; *p* < 0.001)
Reported not receiving medical care**	Unadjusted	Ref	1.54 (1.67–2.03; *p* = 0.002)	2.65 (1.73–4.06; *p* < 0.001)
	Adjusted^1^	Ref	1.37 (1.03–1.83; *p* = 0.03)	2.08 (1.27–3.39; *p* = 0.003)

Notes: Odds ratios were estimated for multinomial logistic regression models with levels of diabetes distress as the outcome (reference = no diabetes distress) and healthcare disruptions as the exposure

* Reference: Did not report delayed medical care

**Reference: Did not report not receiving medical care

^*1*^ *Adjusted for age, sex, race and ethnicity, education, income, health insurance status, marital status, employment status, geographical region, anxiety, and depression*

### Sensitivity analysis

The results of the primary analysis were confirmed when multinomial logistic regression was conducted with the original five-level categorization of diabetes distress, which showed a higher odds of experiencing each level of diabetes distress (always, usually, sometimes, and rarely) among individuals with Type 1 and Type 2 diabetes who reported delayed medical care or not receiving medical care (Supplementary Table 1). This sensitivity analysis confirms that findings from the three-level categorized diabetes distress variable and the five-level reported frequency of diabetes distress offer similar interpretations.

## Discussion

We conducted an analysis of diabetes distress associated with pandemic-related disruptions to healthcare in 2021 using nationally representative data. We found a higher odds of moderate or high diabetes distress among adults who reported experiencing delayed or missed medical care due to the pandemic. These findings can be generalized to the US population, and adds more recent data to the experiences of individuals with diabetes during the pandemic.

The prevalence of diabetes distress was high among adults with diabetes. Over half of individuals with Type 1 or Type 2 diabetes reported experiencing high or moderate diabetes distress during 2021. The prevalence of COVID-19 pandemic-related healthcare interruptions was also substantial. Under a quarter of individuals with either Type 1 or Type 2 diabetes reported experiencing delayed or missed medical care within the past year.

Our findings show that heightened distress due to pandemic-related impacts on healthcare persisted in 2021, even after COVID-19 vaccines were available and social distancing policies were relaxed. National prevalence data for diabetes distress prior to the pandemic is not available; however, subpopulation studies have found the pre-pandemic prevalence of diabetes distress to range from 8 to 42.1% among adults with Type 1 diabetes and 27.4–51.3% among adults with Type 2 diabetes [4, 37–39]. These prevalence ranges are similar to the prevalence of combined high and moderate diabetes distress found by our study for Type 1 and Type 2 diabetes. Our study found that adults with Type 1 diabetes were 3 to 4 more likely to experience moderate or high diabetes distress if they reported delayed medical care and roughly 3 to 3.5 times more likely to experience some or high diabetes distress if they reported missed care. Adults with Type 2 diabetes were approximately 1.5 or 2 times as likely to report moderate or high diabetes distress, respectively, if they reported delayed or missed medical care. The findings suggest that impeded access to healthcare during the pandemic, particularly among people with Type 1 diabetes, was a source of stress. This may inform recommendations for diabetes care during future public health emergencies.

We hypothesized that the pandemic may be associated with increased diabetes distress through pandemic-related healthcare interruptions. Our findings show that individuals who reported delayed or missed medical care had a statistically significant higher odds of diabetes distress regardless of diabetes type. This supports our hypothesis and suggests that the psychological burden of pandemic-related healthcare changes on individuals managing their diabetes was significant and should not be overlooked. Inconsistent health care access could have posed a risk for individuals with diabetes during the pandemic.

This study has many strengths. First, nationally representative data were used, so findings can be generalized to the entire US population with Type 1 or Type 2 diabetes. Second, the most recent yearly data (2021) were used. Third, the dataset that was analyzed measured many covariates associated with healthcare access and diabetes distress, and these covariates were included in the regression models. Fourth, a sensitivity analysis was conducted, which validated the findings that there was a higher odds of diabetes distress among individuals who experienced pandemic-related healthcare interruptions, regardless of how diabetes distress was defined. Fifth, interaction was assessed between healthcare access and diabetes type.

This study has some limitations. First, the data used are cross-sectional, so conclusions cannot be made about causation between the pandemic-related healthcare interruptions and diabetes distress. It is possible that individuals experiencing diabetes distress may be more likely to delay or not seek medical care. Second, all data are self-reported and collected a single time point. Individuals who are experiencing diabetes distress may be more likely to recall or report failures to access to care. This could lead to recall and social desirability biases for the exposures and issues of subjectivity in the outcome variable, as diabetes distress was not measured objectively using a diagnostic tool. Third, case-wise deletion of observations with missing values for covariates could have created bias in the estimates from the regression analyses. Among adults with Type 1 diabetes, 8.4% were excluded due to missing data and among adults with Type 2 diabetes, 4.3% were excluded due to missing data. Fourth, the use of a single-item measure of diabetes distress may have more measurement error than other multiple item measures such as the widely used Diabetes Distress Scale [40]. Nevertheless, the measure adds value to our understanding of perception of diabetes distress nationally.

In order to gain a better understanding of the nature of association, it could be beneficial to investigate how health care access and diabetes distress levels may have changed or persisted from pre-pandemic to present. Future research can also look at other potential risk and protective factors of diabetes distress during the pandemic, such as social support changes during the pandemic. For example, phenomena, such as transportation, work schedules, caregiving, and social support were all affected by the pandemic. It is possible that limitations in transportation and mobility, competing demands of work or caregiving, or reduced social support could have impeded an individual’s ability to access health care and level of diabetes distress. Studies with a larger sample size for Type 1 diabetes may be beneficial to reduce bias.

These findings expand on early pandemic research on diabetes distress and suggest the need for the consideration of the psychological impact of the pandemic on individuals managing their diabetes. As the US shifts to a post-pandemic reality, it could be beneficial to investigate whether individuals with diabetes are returning to and remaining engaged with health care, either in-person or telemedicine, and whether diabetes distress levels have normalized among individuals with diabetes. It could also be beneficial to explore approaches to improving routine accessibility to healthcare providers for individuals with diabetes.

## Conclusion

We found that pandemic-related health care interruptions persisted in 2021 and prevalence of diabetes distress was substantial in 2021, even after COVID-19 vaccines were available and social distancing policies were relaxed. Impeded access to healthcare, in both adults with Type 1 and Type 2 diabetes, appear to have been a source of stress. Consistent availability of health care services may be beneficial to mitigating diabetes distress during public health emergencies like the COVID-19 pandemic.

### Electronic supplementary material

Below is the link to the electronic supplementary material.

Supplementary Material 1

## Data Availability

The dataset analyzed in this study is publicly available in the NHIS repository, https://www.cdc.gov/nchs/nhis/2021nhis.htm.

## Abbreviations

NHIS: National Health Interview Survey
US: United States
