# Supplementary material for: COVID-19 pandemic-related healthcare interruptions and diabetes distress: a national study of US adults with diabetes
Source: BMC Public Health. 2024 Feb 16;24:493. doi: 10.1186/s12889-024-17921-3 (PMC10870610; doi:10.1186/s12889-024-17921-3)
Supplement: Supplementary file 1 — Supplementary Material 1 [file 12889_2024_17921_MOESM1_ESM.docx]

**Supplementary Table 1. Polytomous logistic regression analysis of pandemic-related health care interruptions and frequency of diabetes distress (sensitivity analysis)**

|  |  | **Individuals with Type 1 diabetes (n=228)** | |
| --- | --- | --- | --- |
|  | **Unweighted frequency of distress levels** | **Unadjusted OR**  **(95% CI)** | **Adjusted^1^ OR**  **(95% CI)** |
| **Experienced delayed medical care (exposure, ref = no)** | | | |
| **Diabetes distress (outcome)**  Never  Rarely  Sometimes  Usually  Always | 12  14  14  7  10 | Ref  3.42 (1.36-8.61)  5.07 (1.90-13.5)  4.08 (1.21-13.7)  4.23 (1.50-11.9) | Ref  3.42 (1.31-8.88)  5.26 (1.94-14.2)  6.11 (1.27-29.3)  2.80 (0.77-10.2) |
| **Did not get medical care (exposure, ref = no)** | | | |
| **Diabetes distress (outcome)**  Never  Rarely  Sometimes  Usually  Always | 10  8  13  3  10 | Ref  2.02 (0.66-6.19)  4.59 (1.61-13.1)  1.95 (0.44-8.64)  5.32 (1.78-15.9) | Ref  1.88 (0.56-6.32)  5.40 (1.74-16.7)  1.98 (0.25-15.7)  4.03 (0.87-18.6) |
|  |  | **Individuals with Type 2 diabetes (n=2534)** | |
|  | **Unweighted frequency of distress levels** | **Unadjusted OR**  **(95% CI)** | **Adjusted^1^ OR**  **(95% CI)** |
| **Experienced delayed medical care (exposure, ref = no)** | | | |
| **Diabetes distress (outcome)**  Never  Rarely  Sometimes  Usually  Always | 197  123  123  29  43 | Ref  1.61 (1.20-2.16)  1.83 (1.36-2.46)  3.03 (1.75-5.23)  2.36 (1.46-3.83) | Ref  1.61 (1.20-2.16)  1.67 (1.21-2.31)  2.52 (1.32-4.80)  2.10 (1.29-3.41) |
| **Did not get medical care (exposure, ref = no)** | | | |
| **Diabetes distress (outcome)**  Never  Rarely  Sometimes  Usually  Always | 139  84  94  23  34 | Ref  1.35 (0.97-1.90)  1.74 (1.24-2.44)  3.28 (1.85-5.79)  2.30 (1.35-3.93) | Ref  1.29 (0.92-1.81)  1.47 (1.02-2.12)  2.50 (1.27-4.91)  1.83 (1.02-3.27) |

*^1^Adjusted for age, sex, race and ethnicity, education, income, health insurance status, marital status, employment status, geographical region, anxiety, and depression*
